# Novel immune–risk score of gastric cancer: A molecular prediction model combining the value of immune–risk status and chemosensitivity

**DOI:** 10.1002/cam4.2077

**Published:** 2019-04-03

**Authors:** Shijie Duan, Pengliang Wang, Funan Liu, Hanwei Huang, Wen An, Siwei Pan, Xin Wang

**Affiliations:** ^1^ Department of Surgical Oncology The First Affiliated Hospital of China Medical University Shenyang China

**Keywords:** chemoradiotherapy and chemotherapy, gastric cancer, immune cell infiltration, immune–related genes, immune–risk status, molecular prediction model

## Abstract

Gastric cancer is still one of the most common and deadly malignancies in the world. Not all patients could benefit from chemotherapy or chemoradiotherapy due to tumor heterogeneity. Therefore, identifying different subgroups of patients is an important trend for obtaining more effective responses. However, few molecular classifications associated with chemosensitivity are based on immune–risk status. In this study, we obtained six key immune–related genes. Using these genes, we constructed a molecular model related to immune–risk status and calculated an individual immune–risk score. The score showed great efficiency and stability in predicting prognosis and identifying different subgroups where persons could benefit from postoperative adjuvant therapy. The patients could be divided into different risk groups based on the immune–related score. For patients in the low–risk group, both postoperative chemoradiotherapy and chemotherapy could significantly improve prognosis on overall survival (OS) and disease–free survival (DFS) (DFS, *P* < 0.001 and *P* = 0.041, respectively; OS, *P* < 0.001, *P* = 0.006, respectively) and chemoradiotherapy was significantly superior than simple chemotherapy (DFS, *P* = 0.031; OS, *P* = 0.027). For patients with an intermediate–risk score, postoperative chemoradiotherapy showed a statistically significant survival advantage over no anticancer treatment (*P* = 0.004 and *P* = 0.002, respectively), while chemotherapy did not. Compared with no adjuvant treatment, neither postoperative chemoradiotherapy nor chemotherapy made significant difference for patients in the high–risk group. Combining the value of immune–risk status and chemosensitivity, the immune–risk score could not only offer us prognostic evaluation and adjuvant treatment guidance, but also improve our understanding about the binding point between chemotherapy or chemoradiotherapy and the immune system, which may be helpful for further expanding the application of immunotherapy.

## 1 INTRODUCTION

Gastric cancer (GC) is one of the most common malignancies in the world.^1^ Although its incidence has continually decreased, it is still ranked as the fifth most common malignancy in the word, especially in East Asian countries (mainly in China).^2^ Its prognosis is relatively poor and is ranked as the third leading cause of cancer death with the 5‐year survival rate being less than 40% in most countries, which mainly results from difficulties in early diagnosis, low rates of radical resection and unsatisfactory effects of adjuvant therapy.^1^, ^3^

Radical surgery remains the only cure for patients with GC.^4^ However, most patients are diagnosed at an advanced stage, for whom simple surgical strategies cannot meet the requirement for the treatment.^5^ Recently, a great number of researchers have devoted to the study about application of adjuvant chemotherapy for GC and have obtained several significant conclusions. The phase III trial, SWOG9008/INT‐0116, established the status of standard treatment for postoperative chemoradiotherapy.^6^ Thereafter, the ACTS GC trial and CLASSIC trial provided strong evidence for the application of postoperative chemotherapy.^7^, ^8^ Therefore, for locally advanced tumors, radical surgical resection with subsequent perioperative chemotherapy or chemoradiotherapy seems to be the main treatment strategy.^9^ Unfortunately, the overall effects of chemotherapy and chemoradiotherapy for patients with GC are relatively unsatisfactory due to tumor heterogeneity.^10^ In addition it is difficult to avoid the additional side effects resulting from ineffective treatment strategies, including immunosuppression, bone marrow suppression and so on, which could worsen the condition and affect the prognosis.^11^ Thus, it is very essential to understand the molecular heterogeneity characteristics of GC and establish a molecular model to classify GC patients into different subgroups, which could be considered in quickly and accurately shaping individualized clinical treatment decisions.

It is known that various components of the immune system are involved in cancer occurrence and development.^12^ The disorder of the immune system in tumor microenvironment is the main factor allowing tumor cells to evade immunologic surveillance and destruction, which has been viewed as a trigger for cancer.^13^, ^14^ However, few molecular classifications associated with the chemosensitivity of GC are based on immune–risk status.^15^, ^16^ Furthermore, the normalization of immune microenvironment has an effect on improving other antitumor therapy, including chemotherapy, radiotherapy and targeted therapy.^17^ And numerous immune–related genes (IRGs) have been reported to be related to the sensitivity of various chemotherapeutic drugs.^18^ However, at the genetic level, the pattern of interactions between the immune microenvironment and chemotherapy remains to be further explored. Therefore, by constructing the molecular prediction model based on IRGs, we could identify the GC population benefiting from adjuvant therapy and further understand the underlying mechanism of interactions between the immune microenvironment and adjuvant therapy, which may be helpful to further expand the application of immunotherapy.

In this study, we measured the expression level of IRGs based on gene expression microarray data and obtained some key IRGs with significant prognostic value. Using these key IRGs, we constructed a genetic prognostic score to predict the prognosis of patients who received chemoradiotherapy. With the genetic prognostic score, we could classify the GC patients into different subgroups and evaluate the effect of chemoradiotherapy in each subgroup.

## 2 METHODS

### 2.1 Microarray data and clinical cohort

The gene expression profile, GSE26253, was downloaded from the GEO database (http://www.ncbi.nlm.nih.gov/geo/), which is a public functional genomic data repository. GSE26253 included a total of 432 gastric adenocarcinoma cases of stage IB to IV (T2bN0 and T1N1, but not T2aN0), according to the 6th American Joint Committee on Cancer/International Union Against Cancer (AJCC/UICC) staging system. These patients were all treated with standard chemoradiotherapy after curative resection and they all had not received neoadjuvant chemoradiotherapy (ACRT) or neoadjuvant chemotherapy (ACT). The details of the clinical and pathological annotations as well as the treatment records are all presented elsewhere. The processing of the samples and measurement of mRNA could also be searched in the GEO database. Overall, we included 432 patients in the development cohort.

**Figure 1:**
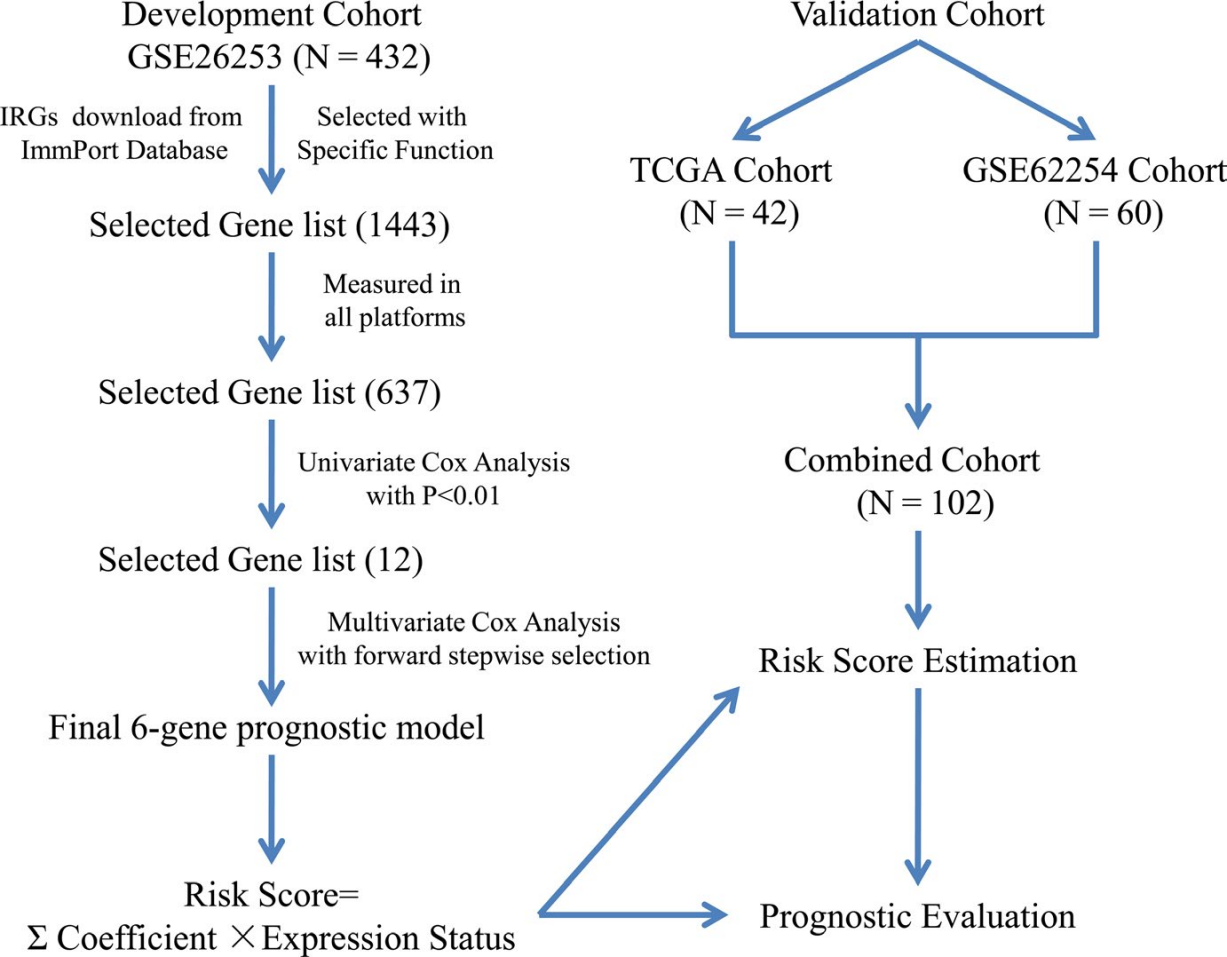
Flow chart of the development and validation of the immune–related–genes prognostic model for patients who received 5–Fu–based chemoradiotherapy after curative resection. IRGs, Immune–Related Genes

To ensure the independence and feasibility of validation, we selected a combination of The Cancer Genome Atlas (TCGA) cohort and GSE62254 cohort to form a validation Cohort. Similarly, the included criteria were set as follows: gastric adenocarcinoma; stage of IB to IV according to the 6th AJCC staging system, without distant metastasis; and standard chemoradiotherapy (combination radiotherapy and fluorouracil and/or platinum) after curative resection. To exclude interference from preoperative treatment and ensure comparability, patients receiving any neoadjuvant anticancer therapy were excluded. Finally, the validation cohort consisted of a total of 102 cases, 42 cases from TCGA and 60 cases from GSE62254. The overall study design is shown in the Figure 1.

### 2.2 Individual prognostic signature based on IRGs

The individual prognostic signature was built based on IRGs, which were obtained from the ImmPort database (https://immport.niaid.nih.gov).^19^ The genes of cytokines, cytokine receptors, and those that were related to the signaling pathways of the T–cell receptor, B–cell antigen receptor, natural killer cell cytotoxicity, and antigen processing and presentation were selected. Among these IRGs, only the genes measured by all platforms were included.

### 2.3 Generation of individual risk score

The Univariate Cox Analysis was performed to select IRGs with a significant prognostic value (*P* < 0.01) as the candidates of the individual risk score. Then, for identifying robust IRGs (*P* < 0.05) associated with overall survival (OS) to build the IRGs risk score, these selected IRGs were further refined with forward stepwise selection by taking use of multivariate Cox analysis. At the same time, a molecular model was constructed, and an individual risk score was calculated based on a combination of coefficients and expression status of these refined IRGs. The risk score was set as R*_i _*= $\sum_{k=1}^{n}\beta_{k}\times x_{i}$, where R*_i _*is for the risk score of *i*th patient, n is for the number of these refined IRGs, β_k_ is for the coefficient of *x_i _*and *x_i_*is for the expression status of IRG *i*. The expression status of IRG is 0 if its individual expression level is lower than the median value; otherwise, the expression status of IRG is 1.^20^ With the median of IRGs risk score, the cohort was stratified into high– and low–immune–risk.

### 2.4 Statistical analysis and immune–related infiltration analysis

The statistical analyses in this study were all conducted in R, version 3.3.1 (https://www.r-project.org/). We compared the OS and disease–free survival (DFS) between different immune–risk score (low– and high–immune–risk score) in the development cohort. The time–dependent receiver operating characteristic (ROC) curves at 5 years were drawn to identify the predictive value of the risk score. Similarly, the OS and DFS between the low– and high–immune–risk score in the combined cohort and the ROC curves at 5 years were drawn to validate the predictive value of the risk score. Furthermore, univariate Cox proportional hazards regression was used to estimate hazard ratios (HRs) between the low– and high–immune–risk scores within different clinicopathological characteristic subgroups in the validated cohort. The survival differences of OS and DFS were all calculated by the log–rank test.

The frozen tumor tissues in TCGA were divided into three parts: top and bottom sections for pathological examination and middle for genomic data. Therefore, we compared immune–related infiltration information, such as lymphocytes, monocytes, neutrophils and necrosis percentage, among the different immune–risk groups using the Wilcoxon rank sum test.^21^

### 2.5 Clinical treatment strategy analysis

To search the guiding value of the risk score in individualized clinical decision making, we further conducted log–rank test among the low–risk, intermediate–risk and high–risk groups stratified by the risk scores with the middle two quarters combined. Kaplan–Meier curves were performed to comprise OS and DFS among the three risk groups treated with different treatment strategies, including chemoradiotherapy, chemotherapy and no treatment.

## 3 RESULTS

### 3.1 Composition of the clinical cohort

A total of 958 GC patients were included in our initial study, of which 432 patients came from GSE26253, 253 patients from TCGA and 273 patients from GSE62254. Their detailed features are shown in Table 1. After screening, 432 patients from GSE26253 formed the development cohort, while 42 patients from TCGA associated 60 patients from GSE62254 were integrated into the validation cohort.

**Table 1:** Basic demographic and tumor characteristics of included patients

Clinicopathological characteristics	GSE 26253 Cohort (N = 432)	TCGA Cohort (N = 253)	GSE 62254 Cohort (N = 273)
AJCC 6th TNM Stage			
IB	68	57	30
II	167	87	97
III	130	68	96
IV	67	41	50
T stage			
T1	—	3	0
T2	—	155	176
T3	—	63	82
T4	—	32	15
N stage			
N0	—	75	37
N1	—	120	126
N2	—	44	69
N3	—	14	41
Sex			
Female	—	91	87
Male	—	162	186
Age, y			
<65	—	110	146
≥65	—	142	127
Missing		1	—
Tumor location			
Lower	—	97	143
Middle	—	95	99
Upper		58	28
Whole		—	3
Missing		3	—
Lauren type			
Intestinal	—	204	141
Diffuse/Mixed	—	49	132
Treatment type			
No treatment	—	126	111
5‐Fu–based ACRT	432	42	60
5‐Fu–based ACT	—	71	81
Other treatment	—	14	21

ACRT, adjuvant chemoradiotherapy; ACT, adjuvant chemotherapy; TCGA, the cancer genome atlas.

### 3.2 Definition of IRGs

There were 1443 IRGs downloaded from the ImmPort database, among which 637 IRGs were measured in all platforms. Using univariate analysis, the impact of these IRGs on survival was measured in the development cohort (Supplementary Table S1). Based on a threshold of P values less than 0.01, a total of 12 IRGs related to significant prognosis were selected as further evaluation factors.

### 3.3 Construction and evaluation of the SIRGs score

**Figure 2:**
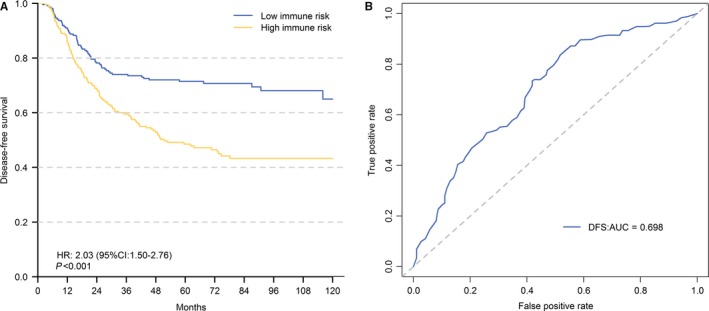
A, Kaplan–Meier Curves of Disease–free Survival stratified by Six Immune–Related Genes (SIRGs) score in high and low risk for patients in GSE26253 cohort. B, Time–Dependent receiver operating characteristic (ROC) curve for SIRGs scores in the GSE 26253 Cohort at 5 Y. AUC, area under curve; DFS, disease–free survival

These 12 IRGs were subjected to multivariate Cox analysis with forward stepwise selection using a Cox proportional hazard regression in the development cohort, and six refined IRGs were selected as the prognostic factors of the prognostic model, including BRD8, CCL25, CMTM3, FPR1, GDF10 and LEPR (Table 2). Then, a six immune–related genes (SIRGs) score was constructed based on the β‐coefficient and expression status of these six genes, by which the development cohort was classified into high– and low–immune–risk groups. Kaplan‐Meier curves of DFS were constructed to assess the prognosis of patients stratified into high– and low–immune–risk groups by the SIRGs score in the GSE26253 cohort (Figure 2A). Compared with the patients in the low–immune–risk, patients in the high–immune–risk group had significantly worse prognoses (HR = 2.03; 95% CI = 1.50‐2.67; *P* < 0.001). Time–dependent ROC curves showed effectiveness of SIRGs score in the GSE 26253 cohort at 5 years (DFS: AUC = 0.698, 95% CI) (Figure 2B). Furthermore, the DFS of patients stratified into the high– and low–immune–risk groups by the SIRGs score presented significant differences for stage I to II patients (Supplementary Figure S1A) and stage III to IV patients (Supplementary Figure S1B) in the GSE26253 cohort.

**Table 2:** Multivariate Cox proportional hazards regression model for disease–specific survival

Predictors		Multivariate analysis			
Gene name	Expression status	β‐coefficient	HR	95% CI	*P* value
BRD8	Low	0	1		
	High	−0.334	0.716	0.528‐0.971	0.031
CCL25	Low	0	1		
	High	−0.398	0.671	0.496‐0.910	0.010
CMTM3	Low	0	1		
	High	0.355	1.426	1.055‐1.927	0.021
FPR1	Low	0	1		
	High	0.409	1.505	1.115‐2.032	0.008
GDF10	Low	0	1		
	High	−0.387	0.679	0.503‐0.918	0.012
LEPR	Low	0	1		
	High	0.460	1.584	1.166‐2.151	0.003

CI, confidence interval; HR, hazard ratio.

### 3.4 Validation of SIRGs score

**Figure 3:**
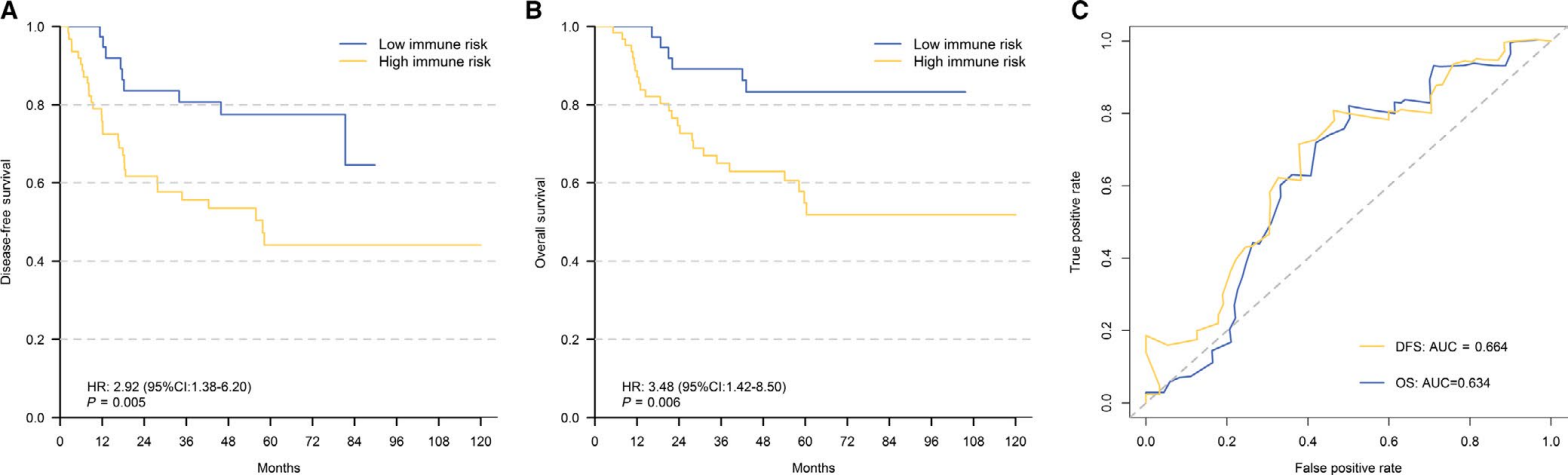
Kaplan–Meier Curves of Disease–free Survival (A) and Overall Survival (B) stratified by SIRGs scores in high and low risk for Patients in combined cohort. B, Time–Dependent ROC Curve for SIRGs scores in the combined cohort at 5 y. AUC, area under curve; DFS, disease–free survival; OS, overall survival

To assess the SIRGs scores, the validation cohort was constructed with two independent clinical cohorts (TCGA cohort and GSE62254 cohort). Similarly, patients in the validation cohort were also divided into high– and low–immune–risk. The survival curves showed that the DFS and OS of the low–immune–risk group had significant survival advantages compared to the high–immune–risk group (DFS: HR = 2.92, 95% CI = 1.38‐6.20, *P* = 0.005; OS: HR = 3.48, 95% CI = 1.42‐8.50, *P* = 0.006) (Figure 3A,B). Furthermore, the ROC curve presented the effectiveness of the risk score regardless of the DFS and OS (DFS: AUC = 0.664, 95% CI; OS: AUC = 0.634, 95% CI) (Figure 3C). The validation indicated great applicability and stability of the SIRGs scores.

### 3.5 Annotation of the SIRGs score

**Figure 4:**
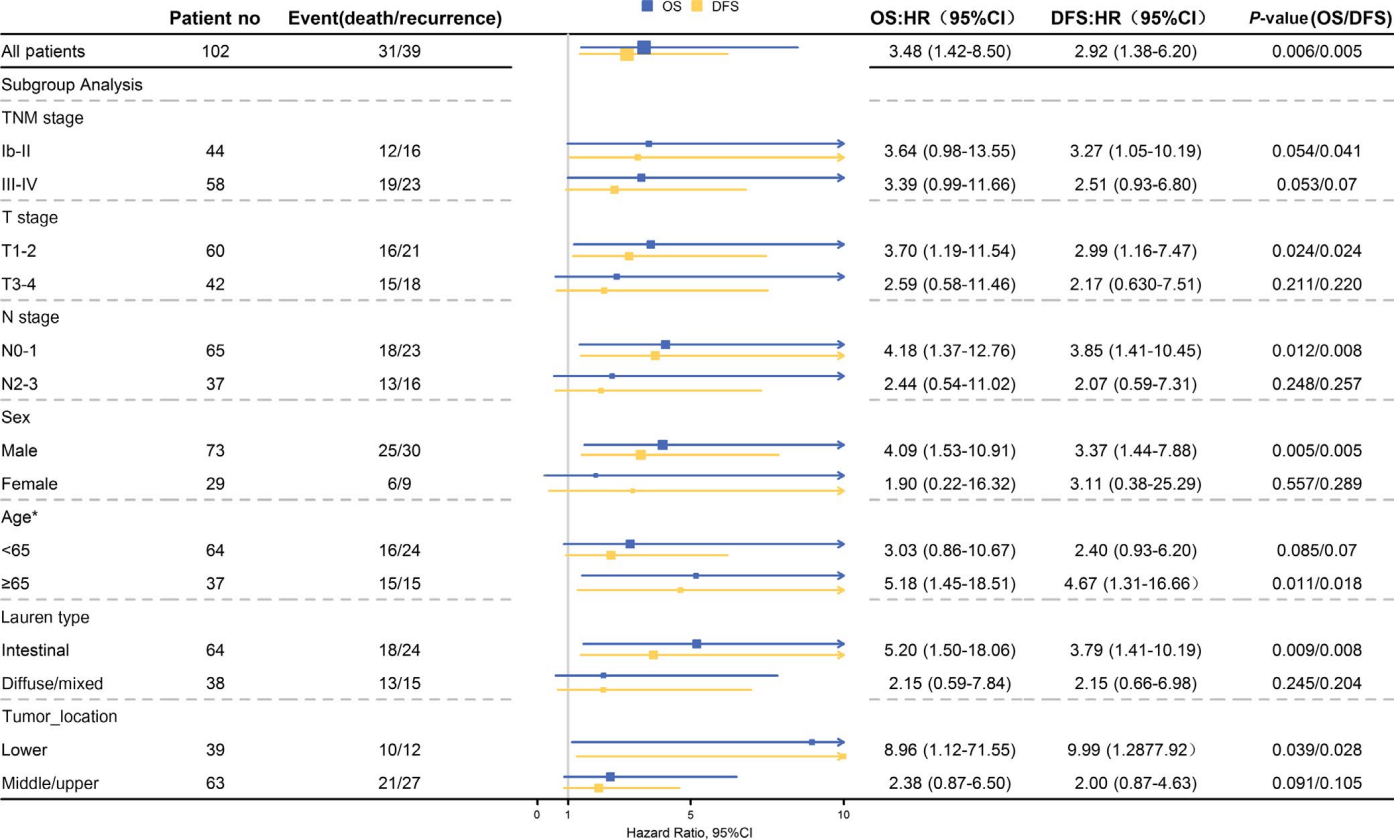
Forest Plot for the hazard ratios (HRs) of High vs Low SIRGs score Risk Groups stratified by different clinicopathological characteristics in combined cohort. DFS: disease–free survival; OS, overall survival. *one patient with age not available were excluded from the analysis

**Figure 5:**
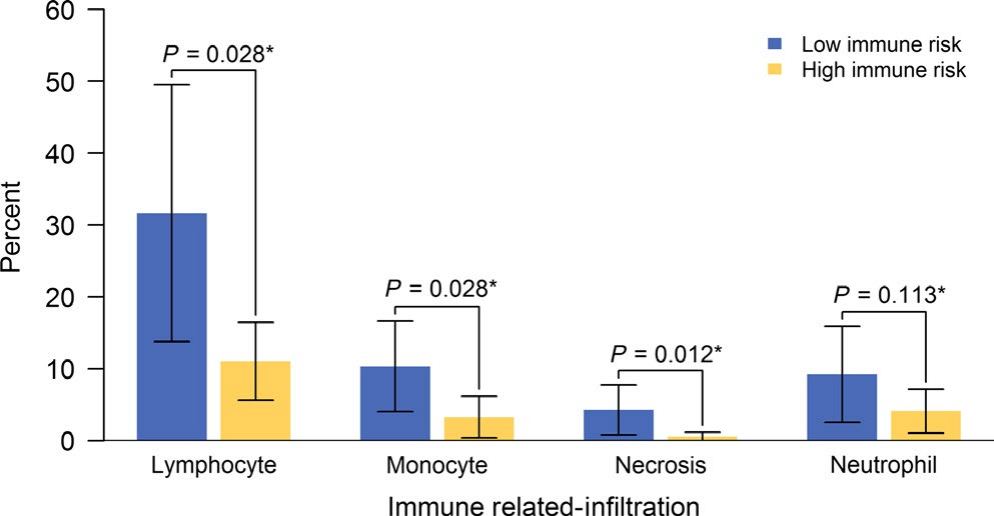
Immune–related infiltration status of high– and low–SIRGs scores in The Cancer Genome Atlas (TCGA) Cohort. *compared the difference between low– and high–immune–risk group by Wilcoxon rank sum test

We also evaluated the prognostic value of the SIRGs score in different subgroups. Based on the SIRGs score, we calculated HRs of high vs low SIRGs score risk groups within subgroups stratified by different clinicopathological characteristics in the combined cohort (Figure 4). It can be seen from the forest plot that patients in groups with low SIRGs score risk had a significantly good prognosis within most subgroups. In order to compare immune–related infiltration status of high and low SIRGs scores, the further analysis was conducted (Figure 5). It could be concluded that the group with low SIRGs scores had a significantly higher infiltration level of Lymphocyte (*P* = 0.028), Monocyte (*P* = 0.028) and Necrosis (*P* = 0.012), but not Neutrophil (*P* = 0.113). Although none of the differences reached statistical significance, patients in the low–immune–risk group had a high infiltration tendency of neutrophil compared with those in the high–immune–risk group. These differences about immune–related infiltration level might elucidate the potential immune mechanism of the SIRGs scores.

### 3.6 Direction of clinical treatment strategy

**Figure 6:**
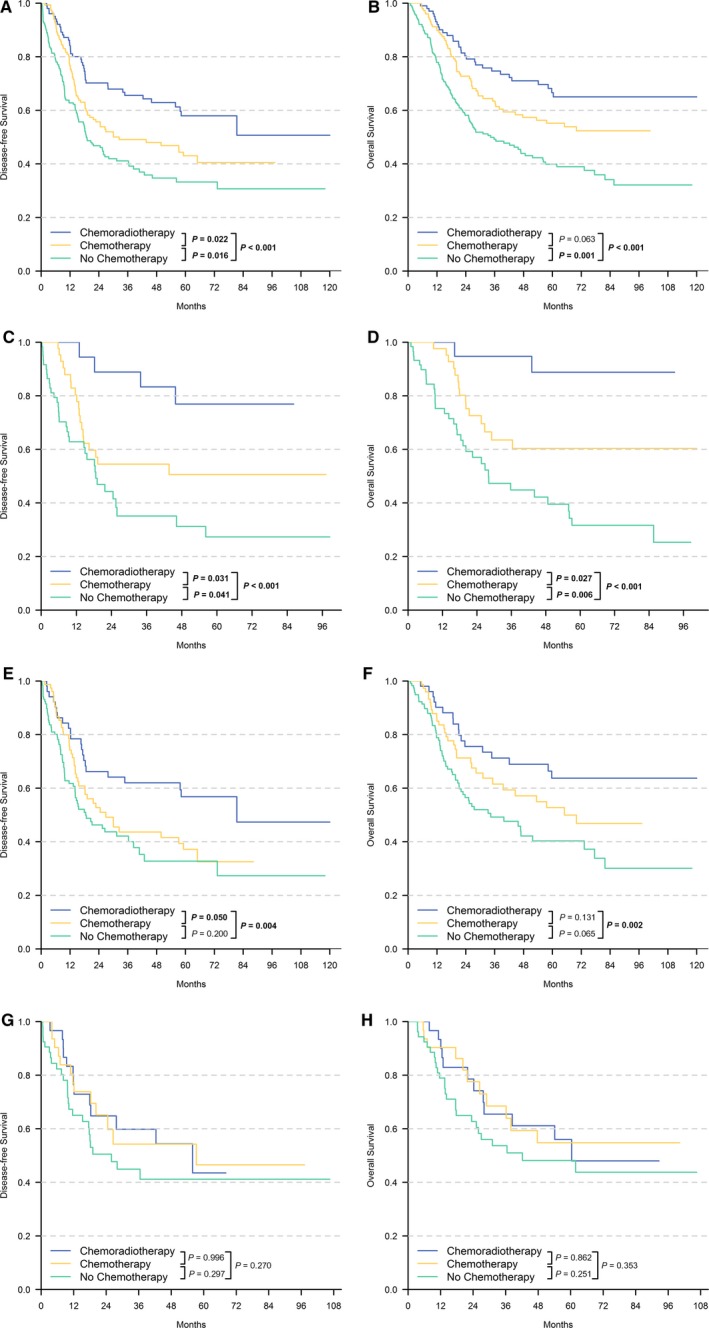
Kaplan–Meier Curves showed the comparison of Disease–free Survival (A, C, E, G) and Overall Survival (B, D, F, H) between different treatment types. All patients in the TCGA and 62254 cohort were combined for analyses (A, B). Combined patients were also stratified into low–risk (C, D), intermediate–risk (E, F) and high–risk group (G, H) according to the risk scores for the analyses

The survival analysis was performed to explore the DFS and OS of different risk groups in the validation cohort treated with different clinical strategies, including chemoradiotherapy, chemotherapy and no adjuvant therapy. All patients in the TCGA and GSE62254 cohort were combined for the analyses (Figure 6A,B). The combined patients were stratified into low–risk (Figure 6C,D), intermediate–risk (Figure 6E,F) and high–risk groups (Figure 6G,H) according to the SIRGs scores for the analyses. For GC patients after curative resection, subsequent chemoradiotherapy or chemotherapy could significantly improve DFS and OS (*P* < 0.001) (Figure 6A,B). Patients in the low–risk group can significantly benefit from chemoradiotherapy (OS, *P* < 0.001; DFS, *P* < 0.001) and from chemotherapy (OS, *P* = 0.006; DFS, *P* = 0.041). In addition, chemoradiotherapy could significantly improve prognosis compared with simple chemotherapy for patients with low–risk scores (DFS, *P* = 0.031; OS, *P* = 0.027). A similar conclusion was not applicable for the intermediate–risk group, for which only chemoradiotherapy could bring significant survival benefits compared with no chemotherapy (OS, *P* = 0.002 and DFS, *P* = 0.040). However, for the high–risk group, patients treated with chemoradiotherapy (OS, *P* = 0.353; DFS, *P* = 0.270) or chemotherapy (OS, *P* = 0.251; DFS, *P* = 0.297) could not obtain any obvious survival benefits compared with no chemotherapy.

## 4 DISCUSSION

GC is still one of the most common and deadly malignancies in the world, whose prognosis is relatively poor.^1^ Most patients are diagnosed at an advanced stage, for whom simple surgical treatment could not yield satisfactory results.^5^ For a long time, whether the application of adjuvant therapy for GC could improve the prognosis was debated. Recently, several clinical trials obtained significant conclusions and established postoperative chemotherapy and chemoradiotherapy as the main treatment strategies for GC.^6^, ^22^ Unfortunately, the benefits from adjuvant therapy for patients were still very limited due to tumor heterogeneity and some additional adverse effects resulting from ineffective treatment strategies could not be avoided.^10^, ^23^ Therefore, it is very critical to identify the benefit population that could benefit from postoperative adjuvant therapy. This study focused on establishing a molecular model to evaluate the prognosis of GC patients and a method to identify populations that could benefit from postoperative adjuvant therapy.

Identifying subgroups of patients who could benefit from specific therapeutic agents has been viewed as an important opinion to obtain more effective therapeutic responses.^24^, ^25^ With advances in molecular research methods, many molecular classifications based on individual differential genetic expressions were established, and appropriate subgroups were identified accordingly. Among these studies, some molecular classifications were revealed to classify patients into different subgroups related to the degree of benefit from postoperative adjuvant therapy.^26^, ^27^ Furthermore, increasing evidences showed that inflammation and the immune system are involved in cancer.^28^, ^29^, ^30^ The immune microenvironment of tumors has been confirmed as a key driver of biological behavior and as a factor that influences chemotherapy.^31^, ^32^, ^33^ However, few classifications could evaluate the prognosis of GC patients based on the risk status of immunological molecules and could be used to guide the application of adjuvant therapy in clinical practice.^15^, ^16^ Therefore, the purpose of this study was to explore a prediction model using the IRGs, by which we could screen GC populations that could benefit from postoperative chemotherapy and chemoradiotherapy.

In the study, we obtained a total of 6 key IRGs, including BRD8, CCL25, CMTM3, FPR1, GDF10 and LEPR, which were all significantly related not only to the immune–risk status but also to prognosis of GC. It had been reported that BRD8, CCL25, FPR1 and GDF10 could be associated with chemotherapy in other types of cancers and could be viewed as novel therapeutic targets to improve the outcome of chemotherapy.^18^, ^34^, ^35^, ^36^ By conducting analysis of these IRGs, we found that they were also associated with the effect of chemotherapy against GC. Based on the SIRGs, we constructed a risk score system and verified its classification value in an independent validation cohort. In a departure from the previous molecular models of GC, the SIRGs score provided us with a stratification standard associated with responses to postoperative chemotherapy and chemoradiotherapy at the genetic level, and this stratification standard was based on the immune–risk status. The SIRGs could be viewed as a prediction model to prejudge the treatment response to different adjuvant treatment strategies in different immune–risk subgroups. Patients in the low–risk group achieved significantly great response from chemoradiotherapy (OS, *P* < 0.001; DFS, *P* < 0.001) and from chemotherapy (OS, *P* = 0.006; DFS, *P* = 0.041). Additionally, chemoradiotherapy could significantly improve the prognosis compared with simple chemotherapy (DFS, *P* = 0.031; OS, *P* = 0.027). For patients in the intermediate–risk group, only chemoradiotherapy could bring significant survival benefits compared with no anticancer therapy (OS, *P* = 0.002 and DFS, *P* = 0.040). For high–risk group, patients did not obtain any obvious survival benefits from chemoradiotherapy (OS, *P* = 0.353; DFS, *P* = 0.270) or chemotherapy (OS, *P* = 0.251; DFS, *P* = 0.297) compared with no anticancer therapy. Based on these significant conclusions, SIRGs could be used as a potential clinical treatment direction at the molecular level. For GC patients with a low–risk score, postoperative chemoradiotherapy and simple chemotherapy were both optional objects, and chemoradiotherapy was the better choice especially for patients with a heavy tumor load. Accordingly, simple chemotherapy was the appropriate choice for patients with intolerant side effects to chemoradiotherapy. For patients in the intermediate–risk group, chemoradiotherapy was the only appropriate choice compared with no anticancer treatment. However, for the high–risk group, both chemoradiotherapy and simple chemotherapy could not improve the prognosis and best supportive care might be worthy of recommendation.

Furthermore, the tumor microenvironment, which is composed of tumor cells, stromal cells as well as cytokines and inflammatory mediators secreted by stromal cells, provides support for tumor biological behavior including occurrence, development, invasion and metastasis.^31^, ^37^, ^38^ These stromal cells mainly consist of fibroblasts, immune cells, inflammatory cells, mesenchymal cells and so on.^32^, ^39^ Increasing evidences has revealed the abnormal infiltration of immune cells and inflammatory cells, such as lymphocytes, monocytes, neutrophils and necrosis, in the tumor microenvironment.^28^, ^29^, ^40^, ^41^ Conducting a correlation analysis of the tissues of tumor sections with different immune–risk scores, we found that the low–immune–risk group had a significantly higher degree of infiltration compared with high–immune–risk group about lymphocyte necrosis and monocyte. Although none of the differences reached statistical significance, patients in the low–immune–risk group had a high tendency toward neutrophil infiltration. These differences about immune–related infiltration levels might elucidate the potential immune mechanism of the SIRGs scores. The results suggest a more intuitive approach for us to determine whether the patient belongs to the high–immune–risk or low–immune–risk group, and this method is simple and feasible.

However, this study has a lot of limitations. Firstly, the sample size of the study was relatively small, which might cause some bias in our conclusions. Secondly, the study was retrospective and the value of this molecular model to predict prognosis was not verified in clinical samples by experiments. Lastly, the development cohort lacked partial clinicopathologic data, and ethnic differences existed between different groups. The potential issues inevitably became a limitation and might result in some statistical bias to the research results. Therefore, we will further conduct the study using raw data from our treatment center and perform further experiments and prospective studies to evaluate the molecular model in clinical practice for our future work.

## 5 CONCLUSION

In conclusion, the SIRGs risk score was a molecular prognostic model related to immune–risk status. It was constructed using the six IRGs and showed certain efficiency and stability in predicting prognosis and identifying different subgroups associated with benefit levels for postoperative chemotherapy or chemoradiotherapy. Compared with simple chemotherapy, postoperative chemoradiotherapy could significantly improve the DFS and OS of patients in the low–risk group. For patients with the intermediate–risk scores, postoperative chemoradiotherapy showed a statistically significant survival advantage over no anticancer treatment, while chemotherapy did not. Neither chemoradiotherapy nor chemotherapy could bring significant survival benefits for patients in the high–risk group.

## CONFLICT OF INTEREST

The authors declare that they have no conflict of interest.
