# Supplementary figures and images for: Novel immune–risk score of gastric cancer: A molecular prediction model combining the value of immune–risk status and chemosensitivity
Source: Cancer Med. 2019 Apr 3;8(5):2675–85. doi: 10.1002/cam4.2077 (PMC6537086; doi:10.1002/cam4.2077)

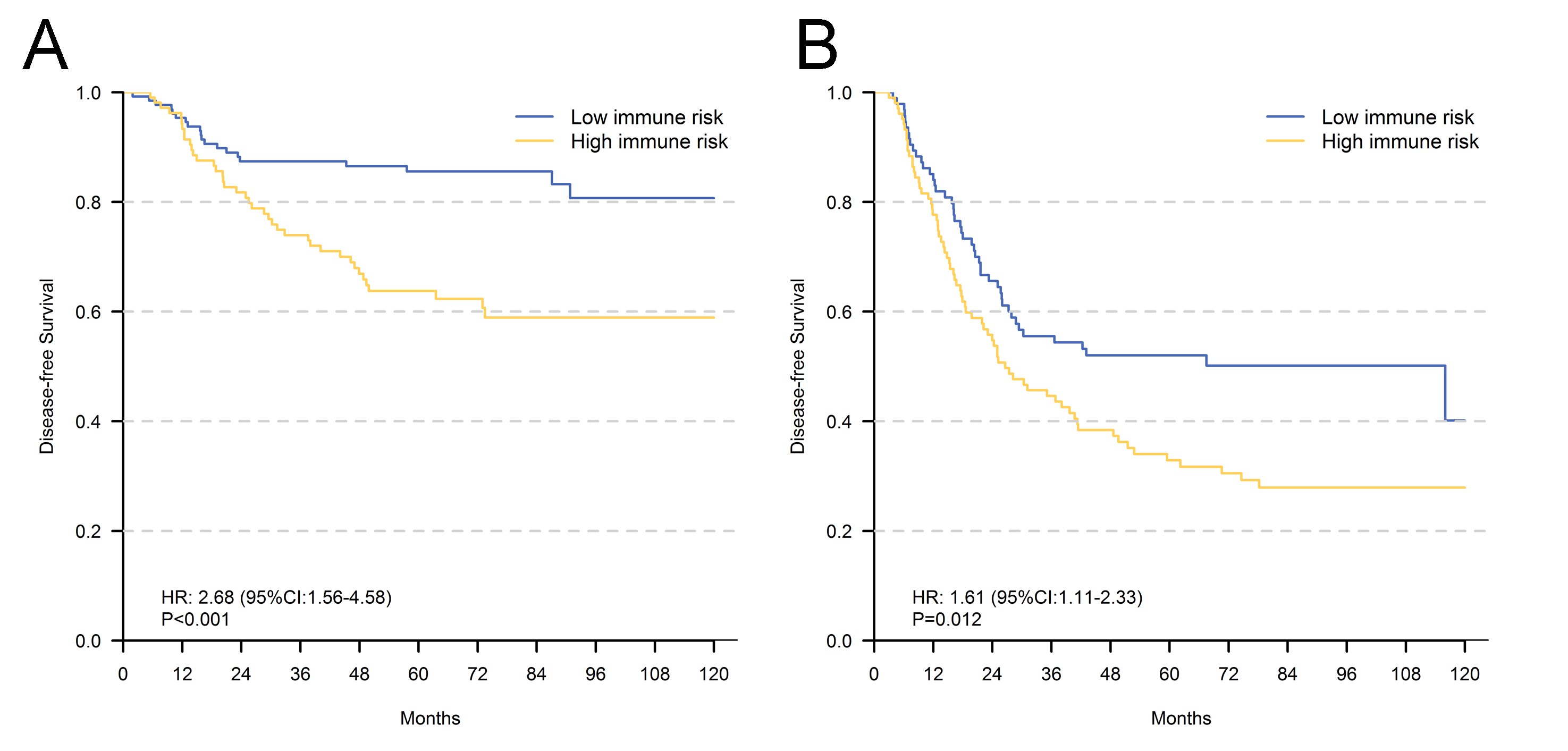

Supplement: Supplementary file 1 [file CAM4-8-2675-s001.tif]
