# Supplementary material for: Novel immune–risk score of gastric cancer: A molecular prediction model combining the value of immune–risk status and chemosensitivity
Source: Cancer Med. 2019 Apr 3;8(5):2675–85. doi: 10.1002/cam4.2077 (PMC6537086; doi:10.1002/cam4.2077)
Supplement: Supplementary file 2 [file CAM4-8-2675-s002.pdf]

Supplementary Table 1: Univariable Cox regression analysis of immune-related genes in developr

Gene list	coefficient	P value	HR	95%CI_Low	95%CI_Upper limit
CD81	-0.52317	0.000651	0.59264	0.438705	0.800587
LEPR	-0.52227	0.00073	0.593173	0.438105	0.803128
TGFB3	-0.46033	0.002669	0.631078	0.46733	0.852202
CMTM3	-0.44755	0.003283	0.639193	0.474297	0.861417
BRD8	0.444026	0.003664	1.558971	1.155485	2.103351
CCL25	0.433554	0.004526	1.54273	1.143668	2.081038
PIK3CA	-0.42793	0.005183	0.651855	0.482889	0.879943
FPR1	-0.40984	0.007181	0.663759	0.492317	0.894905
GDF10	0.407039	0.007523	1.502363	1.114665	2.024909
PSMD6	0.405541	0.007663	1.500114	1.113448	2.021056
RARB	-0.39638	0.009302	0.672753	0.499028	0.906958
NRP1	-0.39337	0.009856	0.67478	0.500523	0.909705
SEMA3B	0.388456	0.010596	1.474702	1.094781	1.986468
LGR5	-0.38822	0.010736	0.678262	0.503346	0.913962
CCL8	-0.38473	0.011432	0.680635	0.505167	0.917052
CHP2	0.380601	0.01217	1.463163	1.086619	1.970191
TNFSF14	-0.37129	0.01427	0.689845	0.512598	0.928382
PTK2B	0.371258	0.014421	1.449557	1.07664	1.951641
FAS	-0.36686	0.016112	0.692909	0.513935	0.934209
CMTM4	0.36471	0.016234	1.440096	1.069633	1.938867
TNFSF8	0.365486	0.016342	1.441214	1.069466	1.942182
CCL24	0.362975	0.016879	1.4376	1.067403	1.936188
VAV2	0.36232	0.017183	1.436658	1.066402	1.935468
GDF11	0.359959	0.017936	1.433271	1.063847	1.930977
PROK2	0.353676	0.019895	1.424294	1.057549	1.918221
LTB4R2	-0.35144	0.020429	0.703673	0.522802	0.947119
VEGFA	-0.34701	0.022161	0.706799	0.52502	0.951516
TNC	0.345387	0.022884	1.412536	1.04904	1.901986
PSMD2	0.345952	0.022995	1.413335	1.048868	1.904448
HGF	-0.34102	0.024853	0.711045	0.527864	0.957795
NR2F1	-0.34152	0.02513	0.710689	0.527068	0.95828
LTBP3	-0.33728	0.026012	0.713712	0.530339	0.960489
NR1I2	0.337478	0.026092	1.401409	1.040995	1.886605
RXRG	0.334146	0.027211	1.396747	1.038316	1.878909
PLXNB1	-0.3332	0.028113	0.716627	0.53226	0.964858
GHR	-0.33191	0.028869	0.717549	0.532802	0.966357
IL17D	-0.33134	0.028987	0.717961	0.533265	0.966627
SEMA3A	-0.32696	0.030663	0.721116	0.536098	0.969986
PDGFRB	-0.32819	0.030704	0.720226	0.5348	0.969944
IL20RA	0.326606	0.031011	1.386255	1.030274	1.865236
TNFSF13B	0.326551	0.031213	1.386179	1.029903	1.865703
PTGDR	-0.32368	0.032351	0.723484	0.537881	0.973131
KIR2DL1	0.322501	0.033261	1.380576	1.025924	1.857829
BTC	0.31861	0.035415	1.375215	1.02199	1.850522
MET	0.314247	0.038433	1.369228	1.016874	1.843674
CSF3R	0.313404	0.038698	1.368074	1.016418	1.841393
IGF1R	-0.31078	0.039896	0.732879	0.544872	0.985756
CSF1R	-0.30985	0.041336	0.733558	0.5447	0.987897
CCR10	-0.30896	0.041608	0.73421	0.545433	0.988323
CRHR1	0.307664	0.042231	1.360243	1.010855	1.830394
NFKBIB	0.306557	0.043052	1.35874	1.009628	1.828567
CXCL5	0.305529	0.043853	1.357342	1.008453	1.826935
GUCA2A	0.305041	0.044004	1.35668	1.008226	1.825564
PRF1	0.30393	0.044784	1.355174	1.0071	1.82355
TNFRSF11	-0.30387	0.044807	0.737955	0.548426	0.992982
CCL13	0.301444	0.046874	1.351809	1.004176	1.819789

AZGP1	0.298963	0.048092	1.34846	1.002512	1.813788
IL6R	0.298527	0.048719	1.347873	1.001679	1.813714
REG1A	0.298728	0.048858	1.348143	1.001497	1.814771
PAK6	0.298461	0.049208	1.347782	1.001035	1.814639
FCGR2B	-0.29707	0.0495	0.742992	0.552396	0.99935
IL23A	-0.29572	0.050347	0.743995	0.553281	1.000448
PPP3CB	-0.29421	0.052037	0.745119	0.553763	1.002599
GRB2	0.294626	0.05204	1.342624	0.997401	1.807336
FGF17	-0.29281	0.053164	0.746168	0.554546	1.004003
LIF	-0.29175	0.053714	0.746954	0.555345	1.004673
NFATC2	0.288767	0.056246	1.33478	0.99234	1.795391
GIP	0.287396	0.057405	1.332952	0.991004	1.792891
CCL18	0.286405	0.058632	1.331632	0.989601	1.791877
NRG1	0.285592	0.058707	1.330549	0.989542	1.789071
PSMD8	0.285264	0.05905	1.330113	0.989157	1.788594
AR	-0.28393	0.060494	0.752821	0.559688	1.0126
DEFA5	0.28265	0.061624	1.32664	0.986336	1.784355
IL10RB	-0.27944	0.06463	0.756209	0.562234	1.017106
NCR1	0.279312	0.065135	1.322219	0.98264	1.77915
CXCL1	0.277963	0.065941	1.320437	0.981855	1.775776
C5AR1	-0.27757	0.066449	0.757626	0.563283	1.019019
FGF2	0.27583	0.068052	1.317624	0.979755	1.772009
CCL26	0.274572	0.069595	1.315967	0.978235	1.770301
THRA	0.273919	0.070171	1.315108	0.97769	1.768975
IFNAR2	-0.27321	0.070273	0.760936	0.566077	1.02287
C3	-0.27359	0.070617	0.760647	0.565425	1.023272
BMPR1A	0.273207	0.070718	1.314172	0.977184	1.767371
RFXAP	0.270815	0.073294	1.311033	0.974774	1.763289
BMP3	0.270401	0.073709	1.31049	0.974396	1.76251
PLXNA1	-0.2694	0.074217	0.763838	0.568269	1.026712
OGN	-0.27064	0.074979	0.762889	0.566347	1.027639
GRP	-0.26941	0.075316	0.763832	0.567623	1.027864
INHBB	-0.2691	0.075568	0.764068	0.567846	1.028094
ICAM2	-0.26784	0.076155	0.765032	0.569018	1.02857
IL1R1	0.267835	0.076616	1.307132	0.971773	1.758224
IL13RA1	-0.26579	0.078878	0.766599	0.569927	1.031139
CXCL13	0.26477	0.079724	1.303131	0.969108	1.752282
CCL16	0.262554	0.082077	1.300247	0.96715	1.748066
CALR	0.261994	0.083639	1.299519	0.965765	1.748612
SEMA6C	-0.25725	0.088453	0.773178	0.575106	1.039468
MLN	0.256338	0.089627	1.29219	0.961119	1.737303
GNRH1	0.25734	0.089954	1.293485	0.960679	1.741585
TNFRSF11,	0.255413	0.091353	1.290995	0.959736	1.736592
CSF1	0.252637	0.095258	1.287416	0.956792	1.732287
PIK3R1	-0.2526	0.095262	0.776779	0.577316	1.045157
XCR1	0.251738	0.095468	1.286259	0.956763	1.72923
VDR	0.25104	0.096201	1.285362	0.956256	1.727734
PTPRC	-0.25061	0.09714	0.778328	0.578857	1.046535
GPI	0.250533	0.097571	1.28471	0.955183	1.727921
IL12RB1	0.250585	0.098004	1.284777	0.954808	1.728779
SYK	0.249689	0.098772	1.283626	0.95432	1.726565
BMPR2	-0.24944	0.099633	0.779238	0.579064	1.048609
PGRMC2	-0.24936	0.099709	0.7793	0.579126	1.048663
SEMA3G	0.24724	0.10165	1.280487	0.952376	1.721638
INHA	0.247207	0.102026	1.280444	0.952073	1.72207
TGFA	-0.24479	0.104932	0.782869	0.582343	1.052444
TNFRSF25	-0.24261	0.107778	0.784575	0.583749	1.054489
CRLF3	-0.24286	0.107923	0.784378	0.583354	1.054675

PIK3R5	0.242517	0.108605	1.274453	0.947689	1.713885
ESRRG	0.241759	0.108948	1.273488	0.947577	1.711493
TOR2A	0.234765	0.119603	1.264612	0.94095	1.699605
TNFRSF10l	0.233936	0.12109	1.263563	0.94004	1.69843
ULBP3	0.233975	0.12142	1.263613	0.939783	1.699027
BDNF	0.233014	0.122843	1.262399	0.938965	1.697241
BRAF	0.231181	0.125881	1.260087	0.937198	1.69422
IL18RAP	-0.23085	0.12611	0.79386	0.590585	1.067102
CD1C	-0.23146	0.126397	0.793377	0.589633	1.067524
CTF1	-0.2302	0.127578	0.794372	0.590779	1.068127
HLA-E	0.229626	0.128354	1.25813	0.93581	1.691466
PAK4	0.227765	0.131814	1.25579	0.933828	1.688757
HLA-F	0.227146	0.133167	1.255013	0.933037	1.688097
LRSAM1	-0.22627	0.133725	0.797506	0.593334	1.071937
PTGER2	-0.22642	0.134066	0.797381	0.592964	1.072266
FGF9	-0.22613	0.134872	0.797613	0.593	1.072828
CD79B	0.225118	0.135748	1.25247	0.931793	1.68351
FGF11	-0.22437	0.137461	0.799021	0.594261	1.074333
FLT4	-0.22356	0.138446	0.799668	0.594939	1.074848
PLAU	-0.223	0.138473	0.800115	0.5957	1.074676
PROCR	0.223568	0.138557	1.250531	0.930283	1.681023
FGF12	0.222321	0.14175	1.248973	0.928447	1.680152
PTGER3	-0.2215	0.142563	0.801317	0.595969	1.077421
IL2RB	0.219915	0.145445	1.245971	0.92667	1.675292
IL18R1	0.218996	0.146737	1.244827	0.926089	1.673267
SEMA4F	0.218337	0.147857	1.244006	0.925546	1.672041
PGF	-0.21788	0.14814	0.804221	0.598591	1.080491
CBL	0.217935	0.148694	1.243506	0.925113	1.671479
NR1H3	0.216853	0.151172	1.242162	0.923811	1.670218
UCN	0.21617	0.151701	1.241313	0.923689	1.668157
PTGDS	0.215471	0.152491	1.240446	0.923401	1.666345
SLIT2	-0.21623	0.152671	0.805553	0.598963	1.083397
IL1RN	0.215103	0.154437	1.23999	0.922237	1.667224
CHUK	0.214184	0.156195	1.238851	0.921396	1.665681
IL17RE	0.213899	0.156318	1.238497	0.921416	1.664694
CCL22	0.2136	0.156971	1.238127	0.921093	1.664283
EDNRB	0.213028	0.15758	1.237419	0.920896	1.662734
SEMA4D	0.213026	0.157986	1.237417	0.920631	1.663209
SOS2	-0.21321	0.158082	0.807986	0.600943	1.086363
GAST	0.212632	0.158657	1.236929	0.920333	1.662434
NR1D2	0.212533	0.158776	1.236807	0.920291	1.662182
EDN1	-0.21288	0.159171	0.808252	0.600948	1.087066
IFNGR2	-0.21034	0.163108	0.810307	0.602942	1.088991
MLNR	-0.2098	0.164893	0.810743	0.602966	1.090116
IL18	-0.20951	0.16576	0.810981	0.603028	1.090646
TNFRSF1B	0.209138	0.165792	1.232615	0.917008	1.656844
TNFRSF1A	-0.20897	0.166273	0.811419	0.603598	1.090794
NR2C2	-0.20821	0.168064	0.812039	0.603959	1.091807
IFNGR1	-0.2078	0.168137	0.812367	0.604519	1.091677
PSMB8	0.207827	0.16954	1.231	0.915121	1.655914
PDGFRA	-0.20788	0.169845	0.812303	0.603687	1.09301
PDGFRL	-0.20689	0.169986	0.813107	0.605079	1.092654
HNF4A	0.206953	0.170283	1.229924	0.914992	1.653254
PDK1	0.206035	0.172154	1.228796	0.914172	1.651703
NCK2	0.205853	0.172439	1.228572	0.914066	1.651292
BMP7	0.205197	0.173887	1.227767	0.913418	1.650299
UBR1	-0.20349	0.176707	0.81588	0.607316	1.096069
EBI3	0.202851	0.178602	1.22489	0.911438	1.64614

PSMD1	0.202057	0.180534	1.223917	0.910575	1.645085
INHBA	-0.20123	0.18243	0.81772	0.608323	1.099197
HAMP	-0.20061	0.183034	0.818234	0.60902	1.09932
FGF20	0.200764	0.183131	1.222336	0.909526	1.642731
PSMD14	0.20083	0.183362	1.222417	0.909355	1.643256
NAMPT	0.199671	0.186232	1.221	0.908102	1.641713
PSMD3	-0.19753	0.189811	0.820758	0.610913	1.102684
PSMD5	-0.19736	0.190892	0.820895	0.610726	1.103388
ESM1	-0.19594	0.19345	0.822061	0.611859	1.104477
AGTR1	0.195996	0.19359	1.216522	0.905296	1.634743
SPP1	-0.19411	0.197379	0.823569	0.61309	1.106306
NR2F2	-0.19361	0.199931	0.823976	0.612826	1.107879
AVPR1A	0.193205	0.199999	1.213132	0.90278	1.630174
STC2	-0.19315	0.200118	0.824361	0.613475	1.107741
RASGRP1	-0.19268	0.201232	0.824746	0.613752	1.108276
IL3RA	0.192658	0.201691	1.212468	0.902041	1.629725
KDR	-0.19247	0.20182	0.824922	0.613847	1.108577
VEGFB	-0.19252	0.202493	0.824876	0.613489	1.1091
IL6	-0.19185	0.203238	0.825432	0.614239	1.109238
CMTM7	-0.19164	0.203407	0.825603	0.614496	1.109236
IGF2R	-0.1915	0.204015	0.825722	0.614477	1.10959
IL1RL1	0.191176	0.204417	1.210673	0.901154	1.626502
FAM3D	0.191045	0.205315	1.210514	0.900691	1.626912
NFATC1	0.190835	0.205452	1.210259	0.900715	1.626184
RORB	0.190522	0.205806	1.209881	0.900661	1.625265
HLA-DQB	0.189789	0.207998	1.208994	0.899743	1.624538
LGMN	0.189718	0.209108	1.208909	0.89913	1.625417
APLNR	-0.18973	0.209112	0.827178	0.615199	1.1122
PSMD7	0.188424	0.211765	1.207345	0.898238	1.622825
TGFBR1	0.187736	0.213421	1.206515	0.897629	1.621693
CHGA	0.187143	0.213737	1.205799	0.897753	1.619546
IL2RG	0.186469	0.215961	1.204987	0.89682	1.619046
BCL10	0.185839	0.217353	1.204229	0.896349	1.617861
FGF5	0.186001	0.217591	1.204423	0.896126	1.618784
GIPR	0.185703	0.218103	1.204065	0.895989	1.61807
PPARG	0.1854	0.218235	1.2037	0.896074	1.616935
LGR6	0.183876	0.222358	1.201867	0.89453	1.614797
IL34	-0.18366	0.223349	0.832216	0.619223	1.11847
MAPK3	-0.18295	0.225429	0.832813	0.619563	1.119463
HRAS	-0.18279	0.22578	0.832947	0.619684	1.119603
PIK3R2	-0.18261	0.226387	0.833094	0.619732	1.119913
TAP1	0.180049	0.233576	1.197276	0.890298	1.610102
PNOC	0.178616	0.235467	1.195561	0.890062	1.605917
NR2F6	-0.1785	0.235955	0.836525	0.622698	1.123777
C3AR1	0.178371	0.236474	1.195269	0.88964	1.605893
CD1A	-0.17749	0.238771	0.837371	0.623269	1.125021
KITLG	-0.17771	0.239551	0.837187	0.622597	1.125738
FGFR3	0.177367	0.239734	1.194069	0.888406	1.604898
GKN1	0.176699	0.240374	1.193272	0.888445	1.602685
CDC42	0.177442	0.240657	1.194158	0.887845	1.606152
IKBKG	-0.17659	0.24143	0.838119	0.623713	1.126229
OXTR	0.175683	0.244376	1.19206	0.886828	1.602348
RAC1	0.175178	0.245356	1.191458	0.88659	1.60116
AKT2	-0.17287	0.251273	0.841245	0.626126	1.130273
MDK	0.172159	0.253514	1.187867	0.883956	1.596265
CD22	0.169645	0.260106	1.184884	0.88196	1.591852
CCL23	-0.16916	0.261859	0.844374	0.628353	1.134662
ADIPOQ	-0.16826	0.264182	0.84513	0.628997	1.13553

PSMD13	0.168274	0.264304	1.183261	0.880573	1.589994
SEMA4G	0.166651	0.268978	1.181342	0.879123	1.587456
IL1RL2	0.166548	0.269397	1.181221	0.878966	1.587413
CCL11	0.165078	0.272576	1.179486	0.878253	1.584037
HLA-C	0.165139	0.273159	1.179557	0.877897	1.584872
TNFRSF13I	0.164368	0.275213	1.178649	0.877323	1.583467
CD3E	0.163425	0.278038	1.177537	0.876463	1.582032
GLP1R	0.163193	0.278767	1.177264	0.876235	1.58171
LYN	-0.16306	0.279051	0.849544	0.632362	1.141318
SSTR2	-0.16295	0.279752	0.849633	0.632275	1.141713
CCL5	-0.16129	0.284751	0.851047	0.633303	1.143657
ITGB2	-0.16091	0.285244	0.851365	0.633782	1.143646
LAT	0.160757	0.286869	1.1744	0.87364	1.578699
ACVRL1	-0.15947	0.289948	0.852594	0.634555	1.145553
EDN3	-0.15913	0.290527	0.852883	0.634945	1.145627
TIE1	-0.15911	0.291205	0.852904	0.634725	1.146079
NR5A2	0.158904	0.291883	1.172225	0.872332	1.575217
MIF	0.157367	0.29596	1.170425	0.87132	1.572206
VAV1	0.156267	0.299597	1.169139	0.87023	1.570718
NFATC3	0.155169	0.30298	1.167856	0.869288	1.568969
RARA	0.155151	0.303022	1.167835	0.86928	1.568928
ROBO3	0.155012	0.303273	1.167672	0.869256	1.568533
CASP3	0.15422	0.305931	1.166748	0.868472	1.567467
FGFR4	0.154375	0.306257	1.166929	0.868176	1.568487
IL4R	-0.15381	0.307937	0.857437	0.637959	1.152423
CXCR5	0.153278	0.308993	1.165649	0.867602	1.566084
PDIA2	0.152358	0.311372	1.164577	0.867087	1.564135
TNFRSF12/	0.152069	0.312543	1.16424	0.866703	1.563921
BTK	0.151151	0.315672	1.163172	0.865803	1.562677
ADRB2	-0.15137	0.315905	0.859525	0.639414	1.155408
CCL28	0.150996	0.316289	1.162992	0.865605	1.562549
PIK3CB	0.150684	0.317669	1.162629	0.865135	1.562424
PSMC1	0.151004	0.317741	1.163002	0.86483	1.563975
GRAP2	-0.15009	0.318854	0.860634	0.640703	1.156059
PDIA3	0.150069	0.319213	1.161914	0.864833	1.561047
SH2D1A	0.149709	0.320449	1.161496	0.864482	1.560556
AGT	-0.14889	0.322795	0.861665	0.641445	1.15749
OSMR	-0.14889	0.323209	0.861665	0.641284	1.157782
NR6A1	0.148748	0.324263	1.160381	0.863284	1.559724
KIR2DL3	-0.14843	0.32494	0.862059	0.641482	1.158483
IL1RAP	-0.14729	0.32787	0.863043	0.642529	1.159238
TNFRSF13(	0.147167	0.328299	1.158547	0.862517	1.556179
TNFRSF14	0.14703	0.329209	1.158389	0.862157	1.556405
MALT1	-0.14679	0.329581	0.863476	0.642827	1.159863
HLA-DOA	-0.1462	0.331834	0.863983	0.643079	1.160768
LTA	0.145604	0.333786	1.156738	0.861	1.554056
BMP1	-0.14509	0.33495	0.864947	0.644029	1.161647
RXRA	0.145017	0.335981	1.156059	0.860367	1.553375
TUBB3	-0.1443	0.338159	0.865624	0.644297	1.162981
NFAT5	-0.14424	0.338948	0.865683	0.644121	1.163458
KRAS	-0.14218	0.34503	0.867469	0.645791	1.165241
PSME1	0.140047	0.352465	1.150328	0.85628	1.545354
CSF2RB	0.139911	0.352501	1.150172	0.85639	1.544734
NRP2	-0.14006	0.352878	0.869302	0.646902	1.168162
CSF2RA	-0.13966	0.353563	0.869653	0.647442	1.168128
VGF	0.139667	0.353909	1.14989	0.855884	1.544892
IL1R2	0.139237	0.355111	1.149397	0.855663	1.543966
MR1	-0.13927	0.355681	0.869995	0.647395	1.169134

GMFG	0.138725	0.357118	1.148808	0.855102	1.543394
CBLB	-0.13845	0.358029	0.870709	0.648123	1.169739
NUDT6	0.137994	0.35963	1.147968	0.854491	1.542241
MICA	0.137252	0.362203	1.147117	0.853866	1.541081
TNFSF15	0.136737	0.363743	1.146526	0.853565	1.540038
CCR5	0.136581	0.364732	1.146347	0.8532	1.540215
RFX5	0.135489	0.368498	1.145096	0.852312	1.538458
PPP3R1	0.135381	0.368844	1.144973	0.852238	1.538259
B2M	-0.13298	0.377046	0.875485	0.651795	1.175943
IL2RA	0.132265	0.380094	1.141411	0.84952	1.533595
GHRL	-0.13224	0.380522	0.876132	0.651946	1.177409
HSPA4	0.131219	0.383238	1.140218	0.848966	1.531388
TAPBPL	-0.13001	0.387852	0.878085	0.653701	1.179489
CAMP	0.130091	0.387918	1.138932	0.847705	1.53021
VEGFC	-0.1297	0.388988	0.878362	0.653915	1.179848
CD70	0.129807	0.389184	1.138609	0.847343	1.529994
S1PR2	-0.12956	0.389263	0.878485	0.654101	1.179841
CD72	-0.12904	0.391202	0.878942	0.654429	1.180477
SDC2	0.128668	0.392512	1.137312	0.846826	1.527443
EGFR	-0.12753	0.397318	0.880268	0.655187	1.182671
NR1H2	-0.12679	0.399761	0.880921	0.6558	1.183321
NR3C1	0.125844	0.403232	1.134106	0.844306	1.523377
ITGAL	-0.12569	0.403648	0.881889	0.656607	1.184466
EDNRA	-0.12555	0.404866	0.882011	0.656408	1.185152
CCR6	0.125505	0.40514	1.133721	0.843681	1.523471
NR4A2	-0.12434	0.40913	0.883079	0.657318	1.186378
ROBO1	-0.1242	0.409445	0.883201	0.657494	1.186389
INPP5D	-0.12415	0.409919	0.883247	0.657415	1.186657
JAG1	0.12376	0.411006	1.131744	0.84258	1.520146
HSPA1L	-0.12339	0.412341	0.88392	0.658105	1.187219
PPP3CA	-0.12261	0.416059	0.884612	0.658309	1.18871
OSGIN1	-0.1225	0.416067	0.884706	0.658545	1.188538
DEFB1	-0.12238	0.416113	0.88481	0.658788	1.188377
VAV3	0.121901	0.417838	1.129642	0.841139	1.517099
IL21R	0.121993	0.418182	1.129746	0.840845	1.51791
FASLG	0.121407	0.420146	1.129085	0.840499	1.516757
CXCL6	0.12112	0.421239	1.12876	0.840262	1.516311
PPP3CC	-0.12089	0.422052	0.886133	0.659679	1.190324
FGFRL1	-0.11877	0.429947	0.888009	0.661189	1.192639
PAK1	0.118386	0.431614	1.125678	0.838067	1.511993
ROBO2	-0.11778	0.434365	0.888893	0.661618	1.194242
TNFSF4	-0.11707	0.436919	0.88952	0.662168	1.19493
TGFBR2	-0.11656	0.438855	0.889977	0.662542	1.195483
AKT3	-0.11646	0.439725	0.890062	0.662394	1.195979
PPARA	-0.11612	0.440208	0.890369	0.662998	1.195716
CYR61	-0.11618	0.440569	0.890314	0.662697	1.19611
LTBP4	-0.11608	0.440711	0.890408	0.662884	1.196025
CTSS	0.115653	0.442094	1.122607	0.835902	1.507648
IL16	0.115611	0.442313	1.122559	0.835839	1.507632
PLCG1	-0.1153	0.443711	0.891098	0.663423	1.196908
PIK3CD	-0.11483	0.445275	0.891518	0.663865	1.197238
CTLA4	0.114435	0.447192	1.12124	0.834735	1.506083
CD4	-0.11418	0.447819	0.8921	0.664319	1.197983
VIPR2	-0.11435	0.448571	0.891942	0.663572	1.198907
PRLR	-0.11383	0.449378	0.892414	0.664483	1.19853
VIP	0.113568	0.45022	1.120268	0.834241	1.504363
PDGFA	0.113373	0.451452	1.120049	0.833832	1.504512
CXCR4	0.111807	0.457442	1.118297	0.832682	1.501879

CXCL2	0.111715	0.457965	1.118194	0.832523	1.501889
THBS1	-0.11182	0.458387	0.894203	0.665382	1.201715
PSMC4	0.111537	0.458736	1.117995	0.832344	1.501678
NPY	-0.11116	0.460552	0.894796	0.666044	1.202113
ANGPTL2	0.110123	0.464082	1.116415	0.831371	1.49919
FGF18	-0.10991	0.466288	0.895911	0.666567	1.204166
ICOS	-0.10908	0.468702	0.896657	0.667552	1.204392
LEP	0.10782	0.473763	1.113847	0.829301	1.496025
PDGFD	-0.10737	0.475857	0.89819	0.668618	1.206585
FAM3C	-0.10695	0.477473	0.898571	0.668959	1.206993
HLA-DPA1	-0.10692	0.477847	0.898593	0.668855	1.207242
IFNAR1	0.106937	0.477907	1.112864	0.828283	1.495223
LIFR	0.105998	0.481236	1.111819	0.82781	1.493268
NMB	0.105996	0.481405	1.111817	0.827719	1.493427
FOS	-0.10563	0.483684	0.899759	0.669506	1.209201
CTSE	-0.10541	0.484479	0.899957	0.669704	1.209374
LTB	0.104417	0.487743	1.110063	0.826534	1.490854
SEMA6D	-0.10428	0.488258	0.900972	0.670871	1.209994
CCL19	0.103217	0.492573	1.108732	0.825647	1.488876
LTB4R	-0.10288	0.494352	0.902232	0.671697	1.211889
HSP90AA1	-0.10199	0.498009	0.903035	0.672333	1.212899
CXCL10	-0.10155	0.499595	0.90344	0.672776	1.213188
CCR7	-0.10127	0.500953	0.903688	0.672869	1.213687
CNTFR	0.101043	0.501676	1.106324	0.823885	1.485587
CCL2	-0.10065	0.504324	0.904247	0.672934	1.21507
LCP2	-0.09858	0.512593	0.906125	0.674596	1.217118
CSF2	0.098234	0.514068	1.103221	0.821328	1.481865
PRKCQ	0.098063	0.514655	1.103032	0.821273	1.481455
RABEP2	0.098053	0.514843	1.103021	0.821181	1.481591
IKBKB	-0.09788	0.515604	0.906757	0.675056	1.217987
CD247	-0.09624	0.522609	0.908245	0.676188	1.21994
ENG	-0.0957	0.525027	0.908732	0.676504	1.220679
IL6ST	-0.09583	0.525482	0.908619	0.675939	1.221394
CD8B	0.095236	0.527326	1.099918	0.818677	1.477775
IL24	-0.09504	0.527456	0.909333	0.677161	1.221107
NR4A1	0.094994	0.528044	1.099652	0.818672	1.47707
LTBR	0.09495	0.528446	1.099603	0.818513	1.477224
HSP90AB1	0.094766	0.529162	1.099402	0.818411	1.476867
BMP4	-0.09458	0.529717	0.909756	0.677362	1.221881
RXRB	0.094535	0.529993	1.099148	0.818323	1.476344
PSMC2	0.093862	0.532785	1.098408	0.817856	1.4752
GCGR	0.093855	0.533196	1.098401	0.817625	1.475596
CLCF1	-0.09355	0.533881	0.910691	0.678211	1.22286
CD19	-0.09341	0.535063	0.910816	0.67802	1.223542
HFE	-0.09278	0.53748	0.911395	0.678631	1.223995
PDGFC	-0.09214	0.541519	0.911982	0.678466	1.225869
HLA-A	-0.09161	0.542471	0.91246	0.679495	1.225298
SDC1	0.091478	0.543198	1.095793	0.815935	1.471638
BMP2	0.090918	0.54555	1.095179	0.81555	1.470685
IL17RA	0.090444	0.547831	1.094661	0.815052	1.470192
PSMC5	0.090233	0.548646	1.09443	0.814952	1.469751
MAP2K1	0.089771	0.550874	1.093923	0.814468	1.469263
PPY	0.089496	0.551975	1.093623	0.814316	1.46873
PRKCA	0.088812	0.55485	1.092875	0.813862	1.467541
ACVR1B	0.087727	0.559843	1.09169	0.812888	1.466116
AMHR2	-0.08774	0.560626	0.916002	0.681644	1.230935
SEMA7A	-0.08651	0.565142	0.917125	0.682983	1.231536
TGFBR3	0.086485	0.565263	1.090335	0.811973	1.464126

TNFRSF19	0.086416	0.565924	1.09026	0.811701	1.464414
RELA	0.086105	0.567351	1.08992	0.811431	1.463989
SEMA4B	0.08612	0.567556	1.089937	0.811274	1.464317
ADRM1	0.085895	0.568902	1.089692	0.810884	1.464364
RASGRP3	-0.08509	0.571829	0.918434	0.683825	1.233535
GDF15	-0.08497	0.572306	0.91854	0.683927	1.233634
JUN	0.084512	0.574111	1.088186	0.810407	1.461178
MAPK1	-0.08451	0.574476	0.918959	0.684182	1.2343
ARTN	0.084431	0.574629	1.088098	0.810247	1.461229
RFXANK	0.084412	0.575015	1.088077	0.810045	1.461537
CYSLTR1	-0.084	0.576837	0.919432	0.68452	1.234961
HBEGF	0.083392	0.5793	1.086968	0.809435	1.45966
SST	0.083191	0.580254	1.086749	0.809248	1.45941
RABEP1	-0.08281	0.582511	0.920525	0.685188	1.236693
TNFSF11	0.082515	0.583521	1.086015	0.808582	1.458638
ERAP1	0.079353	0.59818	1.082587	0.80593	1.454213
ZAP70	-0.07925	0.598201	0.923807	0.687974	1.240482
CANX	-0.07914	0.5989	0.923913	0.68796	1.240792
CXCL17	0.078051	0.603782	1.081178	0.805155	1.451826
IL1B	0.077598	0.605945	1.080688	0.80475	1.451242
PTGER1	0.07754	0.606085	1.080625	0.804789	1.451002
GRN	-0.07744	0.606545	0.925486	0.689262	1.242668
CCL21	0.076734	0.60983	1.079755	0.804143	1.44983
KIR3DL1	-0.07628	0.612081	0.926558	0.689978	1.244257
FLT3LG	-0.07576	0.615041	0.927036	0.690017	1.245472
PIK3CG	0.075457	0.615848	1.078377	0.80308	1.448046
TAP2	0.075445	0.616559	1.078364	0.802631	1.448821
SCG2	-0.0738	0.623699	0.928858	0.691688	1.247351
SEMA6B	-0.07308	0.627474	0.929525	0.69195	1.24867
GNRH2	0.072754	0.628626	1.075466	0.800859	1.444231
RORC	0.071613	0.63407	1.074239	0.799911	1.442647
PRKCG	0.071497	0.634853	1.074115	0.799656	1.442773
PSMC6	-0.07119	0.636035	0.931288	0.6935	1.25061
MAP3K14	0.07115	0.636097	1.073743	0.799657	1.441773
NRG4	-0.07072	0.638214	0.931721	0.693837	1.251164
IL10	0.070285	0.640351	1.072814	0.798863	1.44071
NENF	0.069306	0.64511	1.071764	0.798014	1.439422
NFYA	0.069284	0.645294	1.07174	0.797941	1.439489
ESR2	0.069137	0.645709	1.071583	0.798027	1.438911
CD3D	0.068692	0.648028	1.071107	0.797532	1.438524
FGF10	0.068634	0.648085	1.071045	0.797643	1.438157
PLAUR	0.068174	0.650512	1.070551	0.797116	1.437784
FAM3B	0.068078	0.650764	1.070449	0.797188	1.437378
SCTR	0.067979	0.651535	1.070343	0.796894	1.437625
NR4A3	-0.06741	0.654335	0.934812	0.695942	1.255669
PENK	0.067279	0.65454	1.069594	0.79659	1.436161
TNFSF9	-0.06684	0.656648	0.93534	0.69659	1.25592
CIITA	0.066668	0.657491	1.06894	0.796094	1.4353
PLXNC1	-0.06594	0.661027	0.936184	0.697194	1.257097
SECTM1	-0.06557	0.663161	0.936538	0.697253	1.257941
CD320	0.065454	0.664501	1.067644	0.794265	1.435117
CD8A	0.065252	0.66453	1.067428	0.794808	1.433556
C5	-0.06528	0.664577	0.936805	0.697426	1.258346
UBXN1	-0.06473	0.667381	0.937323	0.697725	1.259201
CXCR3	0.064352	0.668882	1.066468	0.794091	1.432271
CD209	-0.06427	0.669316	0.93775	0.698218	1.259457
FYN	0.063736	0.671804	1.065811	0.793648	1.431307
NR0B2	0.063162	0.674632	1.065199	0.793161	1.43054

CSPG5	-0.06257	0.67758	0.939349	0.699407	1.261605
CD74	0.062384	0.678405	1.064371	0.79255	1.42942
NOV	-0.06171	0.681708	0.940153	0.700033	1.262637
CLEC11A	-0.06054	0.687687	0.941253	0.700662	1.264456
IGF1	-0.06024	0.688926	0.941538	0.701046	1.264531
IL11	0.059333	0.693251	1.061128	0.790187	1.424971
HNF4G	-0.05869	0.696529	0.943	0.702136	1.26649
CNTF	0.057555	0.701915	1.059243	0.78885	1.422319
HCST	-0.05709	0.704242	0.94451	0.703386	1.268293
PSMC3	-0.05699	0.70489	0.944603	0.703335	1.268635
HSPA5	0.056517	0.707068	1.058145	0.788008	1.420887
CD48	-0.05605	0.709611	0.945494	0.703948	1.26992
CCL20	-0.05592	0.710073	0.945615	0.704169	1.269849
LCK	-0.0555	0.712071	0.946015	0.704542	1.270251
CD1D	0.055323	0.71303	1.056882	0.787025	1.419268
CCR1	-0.0549	0.715121	0.946583	0.704907	1.271117
CAT	-0.05475	0.715985	0.946722	0.704908	1.271489
TNFRSF4	0.05469	0.71641	1.056213	0.786322	1.41874
RAF1	0.054536	0.716851	1.05605	0.786478	1.418021
PTHLH	0.053982	0.719666	1.055465	0.785991	1.417328
PTGER4	0.053658	0.721236	1.055124	0.785771	1.416809
CD40	-0.05312	0.724011	0.948267	0.706123	1.273448
PSMD4	0.052888	0.725143	1.054312	0.785105	1.415828
IL17B	0.052837	0.725284	1.054258	0.785166	1.415572
IL15RA	0.052177	0.728638	1.053563	0.784594	1.414738
HLA-DRA	-0.05073	0.735855	0.950535	0.707887	1.276357
AP3B1	0.050411	0.737543	1.051703	0.783151	1.412345
LTBP1	-0.05032	0.738048	0.950921	0.708047	1.277106
EPOR	0.049202	0.743536	1.050432	0.782278	1.410507
PLXNB3	-0.04887	0.745303	0.952309	0.709139	1.278865
NFKBIE	-0.04755	0.751845	0.953565	0.710161	1.280395
ARAF	0.047503	0.752085	1.048649	0.780962	1.408089
KIAA0368	0.047505	0.752092	1.048652	0.780944	1.408129
CXCL16	0.047482	0.752276	1.048628	0.78086	1.408217
CTSG	-0.04705	0.754769	0.954043	0.710168	1.281665
GMFB	-0.04679	0.755781	0.954286	0.710591	1.281554
AKT1	0.046702	0.756236	1.04781	0.780231	1.407154
CXCL12	-0.04452	0.767196	0.956456	0.712288	1.284322
LILRB3	-0.04453	0.767584	0.956445	0.711867	1.285053
CX3CL1	-0.04444	0.767592	0.956536	0.712379	1.284375
OPRL1	0.04433	0.768204	1.045328	0.778432	1.403732
CD79A	-0.04348	0.772511	0.957452	0.713009	1.285698
IL27RA	-0.04346	0.772541	0.957468	0.713073	1.285626
FGF1	-0.04318	0.774103	0.957736	0.713145	1.286217
PSME3	-0.04278	0.775989	0.958121	0.71358	1.286463
MPL	0.042689	0.776548	1.043614	0.777161	1.40142
PTPN11	-0.04254	0.777385	0.958349	0.713578	1.287082
ADM	0.042439	0.777814	1.043352	0.776976	1.401052
AVP	-0.04195	0.780284	0.958913	0.714095	1.287664
TAPBP	0.040922	0.785568	1.041771	0.775786	1.398952
IL7R	0.04086	0.78587	1.041706	0.775758	1.398826
GSK3B	0.040778	0.786263	1.041621	0.775722	1.398663
IL15	0.040274	0.788872	1.041096	0.775297	1.398021
NCK1	-0.0402	0.7892	0.9606	0.715422	1.289802
IL1A	-0.04006	0.789977	0.960729	0.715425	1.290143
KLRC3	0.03979	0.791646	1.040592	0.774593	1.397936
PTN	0.039688	0.79189	1.040486	0.774824	1.397236
TXLNA	-0.03953	0.792812	0.961242	0.715701	1.291023

CCR4	-0.03899	0.795446	0.961765	0.716252	1.291434
CR2	0.037775	0.801636	1.038497	0.773428	1.394411
CRIM1	0.036863	0.806476	1.037551	0.772544	1.393465
PSMD10	-0.03652	0.808164	0.964142	0.717989	1.294684
CD1E	-0.03624	0.809549	0.964409	0.718237	1.294954
ADM2	-0.0362	0.809872	0.964447	0.718133	1.295247
TNFRSF17	0.036165	0.809937	1.036827	0.772167	1.392199
S100A6	0.035758	0.81203	1.036405	0.771858	1.391624
TNFRSF10	0.03551	0.813329	1.036148	0.771646	1.391314
PYY	0.035438	0.813665	1.036073	0.771633	1.391138
HTR3A	0.035209	0.814875	1.035836	0.77142	1.390885
GZMB	0.034812	0.817092	1.035425	0.770903	1.390713
CMKLR1	0.034609	0.818	1.035215	0.770924	1.390111
KIR3DL3	0.033652	0.823019	1.034225	0.770094	1.38895
RHOA	0.033442	0.82401	1.034008	0.770057	1.388433
IFITM1	-0.03344	0.824067	0.967111	0.720172	1.298723
FCGRT	0.033433	0.824352	1.033998	0.769665	1.389113
TRPC4AP	0.033188	0.825296	1.033745	0.769903	1.388003
KL	-0.03301	0.826312	0.967533	0.720499	1.299266
SH3BP2	0.032984	0.826364	1.033534	0.769729	1.387751
CXCL14	-0.03282	0.827297	0.967715	0.720622	1.299533
ESRRA	0.031336	0.834916	1.031832	0.768457	1.385475
HLA-G	0.031295	0.835235	1.03179	0.768274	1.385692
KLRD1	0.030939	0.837004	1.031422	0.768117	1.384986
IL17C	-0.03042	0.839678	0.970041	0.722451	1.30248
MIA	-0.03024	0.840605	0.97021	0.722549	1.302758
NR2C1	0.029896	0.842398	1.030347	0.767355	1.383474
NTF3	-0.02982	0.842822	0.970621	0.722836	1.303345
NDP	0.029548	0.844258	1.029989	0.767016	1.383121
STC1	-0.02926	0.845679	0.97116	0.723282	1.303988
BMPR1B	-0.02894	0.847369	0.971471	0.723485	1.304459
IL10RA	0.028896	0.847613	1.029318	0.766572	1.38212
CTSB	0.028777	0.848317	1.029195	0.766356	1.382181
NFYB	-0.02843	0.85005	0.971967	0.723825	1.305177
HSPA2	-0.02823	0.851134	0.972163	0.72392	1.305531
FLT3	0.027695	0.853881	1.028082	0.765644	1.380475
SHC1	0.02732	0.855846	1.027696	0.765344	1.37998
BMP8B	0.027321	0.855867	1.027698	0.765299	1.380065
UCN3	-0.02601	0.862669	0.974321	0.725585	1.308325
MICB	0.025875	0.86344	1.026213	0.764155	1.378141
UTS2	-0.0257	0.864268	0.974627	0.725881	1.308615
SOS1	0.025216	0.866863	1.025537	0.763689	1.377165
OGFR	-0.0252	0.866918	0.975116	0.726202	1.309347
OSM	-0.02514	0.8672	0.975174	0.726292	1.309341
FGFR2	-0.02421	0.872147	0.976078	0.726809	1.310838
NFYC	-0.02395	0.873524	0.976338	0.727026	1.311143
IL17RD	0.023931	0.873582	1.024219	0.762727	1.375361
SEMA4A	-0.02369	0.874807	0.976588	0.727314	1.311295
FLT1	-0.02329	0.87692	0.97698	0.727594	1.311845
RARG	-0.02324	0.877184	0.97703	0.727633	1.311908
NFKB1	-0.02281	0.87943	0.977449	0.727953	1.312456
SORT1	0.022523	0.880935	1.022778	0.761706	1.373332
SEMA3F	0.022332	0.882012	1.022583	0.761417	1.373329
CXCR6	0.021895	0.884275	1.022136	0.761146	1.372618
BID	0.021651	0.885501	1.021887	0.76106	1.372105
ADIPOR1	-0.02153	0.886228	0.978698	0.728706	1.314454
VIPR1	0.021521	0.886235	1.021754	0.760866	1.372096
HLA-DMB	-0.02134	0.887204	0.978885	0.728896	1.314613

PLCG2	-0.02124	0.887699	0.978988	0.729085	1.31455
CKLF	-0.02097	0.889071	0.979243	0.729268	1.314905
SDC4	0.0207	0.890489	1.020916	0.760353	1.370771
PTPN6	-0.01998	0.894317	0.980221	0.729995	1.31622
FCER1G	0.019754	0.895529	1.019951	0.759497	1.369722
NFATC4	0.019646	0.896053	1.01984	0.759517	1.369387
NRTN	0.019087	0.899042	1.01927	0.758982	1.368824
TNFSF10	0.019075	0.899095	1.019258	0.759002	1.368753
CD3G	0.018903	0.899986	1.019082	0.758901	1.368465
TNFSF13	-0.01889	0.900038	0.981288	0.730791	1.317648
GALR2	0.018694	0.901092	1.01887	0.758725	1.368212
PTAFR	-0.01854	0.901856	0.981631	0.731099	1.318015
LGR4	-0.01833	0.902962	0.981835	0.731229	1.31833
FGFR1	0.017572	0.90698	1.017727	0.757925	1.366585
RELB	0.017548	0.907103	1.017703	0.757912	1.366543
NRAS	-0.0173	0.908391	0.982845	0.731949	1.319742
IL17RC	-0.01705	0.909716	0.983095	0.732179	1.32
PPARD	0.016934	0.910337	1.017079	0.757449	1.365701
CMTM8	-0.01628	0.91377	0.983851	0.732732	1.321032
CDK4	0.016234	0.914202	1.016367	0.756471	1.365553
HLA-DMA	-0.01582	0.916232	0.984301	0.73294	1.321865
IL17RB	0.015442	0.918213	1.015561	0.756318	1.363666
OXT	-0.0154	0.918487	0.984719	0.733218	1.322488
CTGF	-0.01478	0.921928	0.985332	0.733232	1.324109
ITK	0.014095	0.925309	1.014195	0.755335	1.361769
SDC3	0.013704	0.927431	1.013798	0.754874	1.361535
GREM1	-0.01369	0.927548	0.986404	0.734355	1.324962
RORA	0.013086	0.930694	1.013172	0.75442	1.36067
CARD11	0.012667	0.932895	1.012748	0.754136	1.360044
HLA-DQA	-0.01233	0.934646	0.987743	0.735565	1.326376
IL11RA	0.012312	0.934751	1.012389	0.753933	1.359445
CD244	-0.01212	0.935794	0.987955	0.735683	1.326733
TEK	0.011935	0.936759	1.012006	0.753616	1.358989
CALCRL	0.011367	0.939738	1.011432	0.753268	1.358076
RAC2	0.011073	0.941289	1.011135	0.753065	1.357643
CMTM1	-0.01085	0.942495	0.989212	0.736707	1.328263
TGFB2	-0.01046	0.944548	0.989595	0.736988	1.328785
JAG2	0.010279	0.945522	1.010332	0.752344	1.356787
MC1R	0.010036	0.946891	1.010087	0.751818	1.357076
PAK2	-0.00979	0.948118	0.990262	0.737471	1.329705
NR2E3	-0.00972	0.948476	0.990331	0.737551	1.329745
TGFB1	-0.00945	0.949856	0.99059	0.737776	1.330035
TYROBP	-0.00861	0.954358	0.99143	0.738358	1.331243
ANGPTL4	-0.0081	0.957083	0.991934	0.738565	1.332223
TNFRSF18	-0.00808	0.957178	0.991954	0.738642	1.332139
TSLP	0.007511	0.960164	1.007539	0.750347	1.352889
ADIPOR2	-0.00702	0.96278	0.993006	0.739481	1.333449
HLA-DPB1	0.006111	0.967589	1.00613	0.749261	1.351062
MAP3K8	-0.00592	0.968595	0.994097	0.740345	1.334824
TNFRSF9	-0.00497	0.973642	0.995044	0.741053	1.336088
HLA-DOB	0.004765	0.97472	1.004776	0.748304	1.349152
CSF3	0.00449	0.976188	1.0045	0.74802	1.348922
PDGFB	-0.00206	0.98909	0.997946	0.743224	1.339967
NODAL	-0.00182	0.99034	0.998181	0.743381	1.340317
PLXNA3	0.001774	0.990594	1.001776	0.745897	1.345432
HDGF	0.001609	0.991465	1.00161	0.745868	1.345042
CBLC	-0.00158	0.991615	0.998417	0.743046	1.341554
TNFRSF21	-0.00157	0.99166	0.998429	0.743552	1.340675

IL32	0.00077	0.995917	1.00077	0.74525	1.343898

nent cohort
